# Interaction between a Martian Regolith Simulant and Fungal Organic Acids in the Biomining Perspective

**DOI:** 10.3390/jof9100976

**Published:** 2023-09-28

**Authors:** Michele Vezzola, Solveig Tosi, Enrico Doria, Mattia Bonazzi, Matteo Alvaro, Alessio Sanfilippo

**Affiliations:** 1 Department of Earth and Environmental Sciences, University of Pavia, Via Ferrata 1, 27100 Pavia, Italy; solveig.tosi@unipv.it (S.T.); matteo.alvaro@unipv.it (M.A.); alessio.sanfilippo@unipv.it (A.S.); 2 Department of Biology and Biotechnology, University of Pavia, Via Ferrata 9, 27100 Pavia, Italy; enrico.doria@unipv.it; 3 Institute of Geosciences and Earth Resources of Pavia, C.N.R, 27100 Pavia, Italy; mattia.bonazzi@unipv.it

**Keywords:** fungal biomining, acidolysis, metal extraction, Martian regolith simulant, *Aspergillus tubingensis*

## Abstract

The aim of this study was to evaluate the potential of *Aspergillus tubingensis* in extracting metals from rocks simulating Martian regolith through biomining. The results indicated that the fungal strain produced organic acids, particularly oxalic acid, in the first five days, leading to a rapid reduction in the pH of the culture medium. This acidic medium is ideal for bioleaching, a process that employs acidolysis and complexolysis to extract metals from rocks. Additionally, the strain synthesized siderophores, molecules capable of mobilizing metals from solid matrices, as verified by the blue CAS colorimetric test. The secretion of siderophores in the culture medium proved advantageous for biomining. The siderophores facilitated the leaching of metal ions, such as manganese, from the rock matrix into the acidified water solution. In addition, the susceptibility of the Martian regolith simulant to the biomining process was assessed by determining the particle size distribution, acid composition after treatment, and geochemical composition of the rock. Although the preliminary results demonstrate successful manganese extraction, further research is required to optimize the extraction technique. To conclude, the *A. tubingensis* strain exhibits promising abilities in extracting metals from rocks through biomining. Its use could prove useful in future in situ mining operations and environmental remediation efforts. Further research is required to optimize the process and evaluate its feasibility on a larger scale.

## 1. Introduction

The global economy needs an ever-increasing amount of trace metals, including rare earth elements, to maintain current production standards and develop new technologies [1,2]. At the same time, primary resources for metallic elements are being rapidly depleted. Therefore, mining industries are forced to turn to matrices containing limited quantities of such elements, which were previously discarded [3].

Extracting trace elements from waste minerals with low concentrations using conventional techniques is difficult and polluting. For this reason, there is a strong need to develop new mining approaches that can implement metal extraction on natural rocks with trace concentrations, while decreasing the environmental impact of these processes [4,5]. Among these new extraction techniques, those based on microorganisms seem the most promising and are, therefore, increasingly being studied [6]. 

Since their use is limited to sulphide-bearing rocks, bacterial chemolithoautotrophic organisms are already exploited and contribute annually to the extraction of copper, gold, and other precious metals from depleted deposits where other extraction techniques can no longer be applied [7,8].

Fungi, which are heterotrophic organisms, may also be used in biomining. This process is based on the organic acids they produce and can be applied to any rock possessing metals in the form of oxides. In addition to the acid leaching process facilitated by acidogenic fungi, siderophores produced by selected fungal microorganisms are also implicated in the mobilization and sequestration of metals [9]. Siderophores are a subject of scientific investigation due to their capacity to solubilize metal ions and form chelate complexes with them. The bioleaching of minerals could be facilitated by the complexation of metals, potentially enhancing their dissolution. Hence, the supplementary metal sequestration capabilities exhibited by siderophores could potentially play a role in the bio-mining process of ores [10]. 

Despite the number of substrates on which fungal biomining can be carried out being greater than those exploitable by bacteria, fungi require a constant supply of organic carbon, which represents a non-negligible increase in costs compared to the bacterial technique [11].

During metabolism, fungi can produce different organic acids that underpin the biomining process [12]. This type of biomining, called bioleaching, is based on chemical interactions between rock containing metal elements in the form of oxides and the organic acid. Extraction takes place through a succession of chemical reactions, the most important of which are acidolysis and complexolysis [13].

Many studies have been conducted on fungal microorganisms and their production of organic acids, and strains of *A. niger* were chosen for the ability to produce acids such as citric, oxalic, malic, and gluconic acids [14,15,16].

The acid-producing capacity of these strains is precisely what is needed for the biomining process as the protons of the acid are able to initiate acidolysis by interacting with the oxides and detaching metal ions from the rock. The remaining anionic component of the acid is then able to complex the metal ions, causing their precipitation within salts and further stabilizing the reaction. In addition to the acid component, other organic molecules are also useful in the complexation process [17], but being in the minority, they are not discussed here.

Biomining has the advantage of reducing energy consumption, in comparison with pyrometallurgy processes. This represents an advantage for biomining not only on terrestrial material, but also in the framework of future space exploration. In extra-terrestrial life contexts, the access to high-quality mineral reserves could be helped by biomining in the view of efficient and sustainable extractive processes, as energy is considered to be a limiting factor in that environment.

On Mars, the metal extraction process could be conducted on the regolith, which will be the most suitable substrate encountered during the exploration of the planet. This material represents an uncemented layer composed of debris with heterogeneous grain sizes that covers the crust of any rocky body [18]. The regolith is generated as a result of crystallization from magmatic eruptions and further modified and dismembered by meteorite and micrometeorite impacts [19]. Hence, the surface of Mars is likely composed of unconsolidated material from both local (residual) and regional (transported) origins. Indeed, a conspicuous component of the regolith is represented by debris with a dusty texture, distributed on a global scale by aeolian processes [20]. The particle size ranges between 70 and 800 μm, while the dominant particle size is around 100 μm. Thus, material classified as sand prevails on the Martian surface [21].

The possible metal availability in the Martian regolith brought attention to the use of in situ resources (ISRUs) in the perspective of future human colonization [19]. The supply of raw materials directly on the red planet could potentially ensure more sustainable space exploration programs while at the same time contributing to reduce the costs of an extra-terrestrial settlement [22].

One promising methodology for extracting metals from the regolith is biomining. When compared to other techniques, fungal biomining could provide further advantages in terms of resource optimization [23]. Studies also suggest that the possibility of metal extraction from Martian regolith could be linked to bioregenerative life support systems (BLSSs) which are considered essential for future human outposts [24,25]. Indeed, the organic carbon needed by microorganisms could come from waste products from human activity in the extra-terrestrial base [26]. This opportunity has not gone unnoticed by the European Space Agency (ESA), which included biomining within the MELiSSA (Micro-Ecological Life Support System Alternative) project. MELiSSA is an innovation network supporting space exploration based on regenerative systems, ultimately aimed at producing food, water, and oxygen from waste generated during the mission. Biomining could thus be used to extract metals from regolith and at the same time contribute to the sustaining the crew on a spacecraft [27]. 

This work focused on the possibility of exploiting the Mars regolith for in situ resource utilization. The chemical composition of Martian regolith is similar to that of terrestrial basalts with mafic or ultramafic compositions. This material is characterised by low silica (SiO_2_) contents and high concentrations of iron (Fe) and magnesium (Mg) and other metallic elements such as aluminium (Al), calcium (Ca), and titanium (Ti). In addition, vanadium (V), zirconium (Zr), and REEs are present at trace levels [28,29].

With these premises, the present work focused on evaluating the extraction capacity of fungal organic acids through acidolysis and complexolysis using the spent-medium bioleaching technique [30]. This technique involves separating the fungus from the acidified rock and exploiting the mere presence of organic acids in the liquid medium released by the fungus for the biomining process [31]. This method can be potentially possible in the context of Martian rock. We will show that the Martian soil, composed of oxide-rich rocks, could pair well with a fungal biomining process based on the findings on bacteria [32].

The experimental work was carried out in two stages; first, the ability of the strain to produce organic acids was analysed and then the susceptibility of the simulant to biomining processes was evaluated. Experiments concerning the change in the pH and in the acid composition (through HPLC) of the leaching solution, the evaluation from the particle size (using a Mastersizer3000, Malvern Panalytical, Malvern, UK) after biomining treatment and the elemental composition of the regolith (using LA-ICP-MS PerkinElmer, Waltham, MA, USA) demonstrated the susceptibility of the aggregate to the treatment.

## 2. Materials and Methods

### 2.1. Martian Rock Simulants and Fungal Strain

The substrate simulating Martian regolith was acquired from the company “The Martian Garden” “https://www.themartiangarden.com/ (accessed on 1 February 2022)”, and was derived from terrestrial material from the Mojave Desert. The kit containing the regolith is named MMS-1 Mojave Mars Simulant and has a mixed particle size of less than 3.17 mm and a density of 1.25 g/cm^3^. The MMS has a basaltic composition with a relatively homogeneous mineralogical and chemical composition. This material is described as a potential geological simulant of the Mars substrate. Although the seller provides the chemical analysis of the rock, the untreated material was further analysed in this work using the same technique applied to the treated rock.

The fungus tested in this work was a strain of *Aspergillus tubingensis* Mosseray (strain code F4), isolated from soil in the Northern Italy in 2019, that was morphologically and molecularly identified and preserved in the collection of the Mycology Laboratory of the University of Pavia. The *A. tubingensis* species is closely related to *A. niger* species. It is one of the most common species within the *Aspergillus* section *nigri*, along with the species *A. brasiliensis*, *A. acidus*, *A. carbonarius*, and *A. niger* itself [33]. 

Due to its similarity to *Aspergillus niger*, *A. tubingensis* shares the characteristic of a rich organic acid production. However, unlike *A. niger*, *A. tubingensis* exhibits a lower toxin production (it is generally recognised as safe by the American Food and Drug Administration), making it an ideal candidate for future biomining processes that could be applied on an industrial scale or in extra-terrestrial contexts [34,35]. 

### 2.2. Preparation of Spore Suspension

Sabouraud dextrose agar (SAB) was chosen as the growth medium. This medium was used in Petri dish to obtain spores of *A. tubingensis*. The solid growth medium was prepared according to manufacturer’s specifications and then sterilized by autoclaving at 121 °C for 20 min. It was cooled to 60 °C and poured into 10 cm diameter Petri dishes. Within the Petri dishes, the fungus was inoculated under sterile conditions and allowed to grow until sporulation.

The spores of 7-day old cultures were collected by scraping them off the surface of the SAB. The spores were then transferred into a test tube containing sterile water gel (1.5% agar) and vortexed to avoid the formation of aggregates and obtain a homogenous suspension. The number of spores was calculated using a Burker chamber and adjusted to 1 × 10^7^ CFU/mL.

### 2.3. Blue CAS Assay for Siderophores

A blue CAS assay was performed to determine the production of siderophores by the *A. tubingensis* strain. To carry out the test, blue CAS dye was prepared as follows [36]:

1. Solution 1: Dissolve 0.06 g of CAS (Fluka Chemicals) in 50 mL of ddH_2_O.
2. Solution 2: Dissolve 0.0027 g of FeCl_3_-6 H_2_O in 10 mL of 10 mM HCl.
3. Solution 3: Dissolve 0.073 g of HDTMA in 40 mL of ddH_2_O.
4. Mix Solution 1 with 9 mL of Solution 2. Then, mix with Solution 3.

The dye was added to SAB medium using the following the formula: 30 g/L SAB, 15 g/L agar, 900 mL water, and 100 mL blue CAS dye. The medium, after the sterilization, was distributed into Petri dishes. *A. tubingensis* was inoculated from the spore suspension into 3 plates; 2 plates without inoculum were prepared as a negative control and two plates of SAB with 3 inoculum for each plate were also prepared as a positive control to evaluate the potential toxicity of blue CAS_SAB media. All plates were placed in an incubator at 27 °C for 7 days.

### 2.4. Acid Production for Spent-Medium Bioleaching Process

To obtain an organic acid solution, 2 mL of an *A. tubingensis* spore suspension was inoculated into 5 Erlenmeyer flasks containing 100 mL liquid medium named TA-N, which is composed of sucrose 10 g/L; ammonium nitrate 1.5g/L; KH_2_PO_4_ 0.5 g/L; methanol 20 mL/L (added after sterilization); magnesium sulphate 0.2 g/L; ferric chloride 0.05 g/L; and calcium chloride 0.02 g/L [37]. Three additional Erlenmeyer flasks containing 100 mL TA-N medium without fungal inoculum were treated under the same conditions and served as a negative control to assess the pH stability of the medium in the absence of fungus.

The flasks were placed in a rotating orbital incubator set at 120 RPM and 27 °C. After 5 days, the growth phase was stopped, and the pH of the medium was measured. 

To obtain a sterile acid mixture without fungal inoculum inside, successive filtration steps were performed. The growth medium of the 5 Erlenmeyer flasks was combined and the pH was measured. The growth medium was filtered with a vacuum pump through a 0.45-micrometre filter. Subsequently, a second filtration step was conducted under sterile conditions by means of a 0.22-micrometre filter, resulting in a sterile acid solution without fungal mycelia.

### 2.5. Spent-Medium Bioleaching Process and Regolith Recovery

This experiment represents the crucial phase of biomining because the rock simulating Martian regolith is brought into contact with the acidified medium produced by *A. tubingensis* and composed of a mixture of organic acids. The biomining experiment was conducted using a spent-medium bioleaching technique, starting with the acidified liquid medium and adding the regolith to it. 

The bioleaching experiment took place inside flasks in which the acid, separated from the fungus, was brought into in contact with the regolith. Three experimental conditions were set up, each with 2 replicates:

- Regolith + acid: Erlenmeyer flasks containing 100 mL of acidified culture medium and 10 g of regolith. This condition represents the actual biomining experiment. The regolith, in contact with the protons of the acid matrix and the anions of the organic acids, undergoes the processes of acidolysis and complexolysis. Two experimental replicas named TR_A and TR_B were set up.
- TA-N + regolith: Erlenmeyer flasks containing 100 mL of unacidified TA-N medium culture and 10 g of regolith. This condition is a control to assess any changes in the pH of the medium and any changes in the elemental composition of the regolith when it is placed in non-acidic medium.
- Acid: Erlenmeyer flasks containing 100 mL of acidified TA-n without regolith. This experimental condition serves as a control to check the pH and composition stability of the acid and any changes in its composition.

The 6 Erlenmeyer flasks were placed in an rotary shaker incubator set at 120 RPM and 27 °C. The pH in each flask was measured after 0, 7, 14, and 21 days. The pH assessment was carried out under sterile conditions under a laminar flow hood directly in the flask and without removing liquid.

After 21 days, the experiment was ended, and the regolith and liquid were recollected from each flask. The collecting process was conducted for each flask from the regolith + acid and TA-N + regolith conditions. 

The regolith from each Erlenmeyer flask was initially transferred to 50 mL Falcon tubes, with 4 tubes with 25 mL of material from each flask. Each tube was centrifuged at 7000 RPM for 3 min. After centrifugation, the supernatant was collected, and the regolith was suspended in 25 mL of sterile distilled water. This centrifugation and washing process was repeated four times. The washed regolith samples were then dried in a lyophilizer for 48 h.

For the experimental sample of regolith + acid, the supernatant was filtered through a 0.2 µm filter after the first centrifugation and stored. HPLC analysis was performed on this sample to assess the new acid composition after contact with the regolith.

These experiments allowed the recovery of data on pH variation during the biomining process compared with the control samples. Additionally, regolith powders were collected from bioleaching flasks (regolith + acid) and control flasks (TA-N + regolith). Finally, liquid samples were also collected, one of the acid after contact with the regolith and the other of the acid left in the incubator for 21 days. These samples made it possible to evaluate the organic acid composition after the experimental phase, comparing it to the composition of the acid produced by the fungus after 5 days.

### 2.6. Major and Trace Element Concentrations

Major and trace element concentrations were determined on fused glass beads produced by mixing 0.5 g of powdered sample with 5 g of lithium metaborate (LiBO_2_) and lithium tetraborate (Li_2_B_4_O_7_) flux. The fused glass beads were realised using a Nieka electric furnace (Nieka, Quebec City, QC, Canada) in a Pt crucible at 1275 °C. Major elements were acquired using an XRF-EDS Rigaku DE, using natural rocks as the internal standard material. Trace element analyses were performed by LASER ablation–inductively coupled plasma–mass spectrometry (LA-ICP-MS) at the Istituto di Geoscienze e Georisorse (IGG)–Consiglio Nazionale delle Ricerche (CNR), Unit of Pavia, Italy. The probe consisted of a Nd-YAG laser microprobe at 266 nm coupled with an 8900 Triple Quadrupole ICP–MS (Agilent, Santa Clara, CA, USA). During the trace element analysis, the laser probe operated at a 10 Hz frequency and with a spot diameter of 50 µm on the target surface. Helium was used as the carrier gas, which was mixed with Ar downstream of the ablation cell. Data reduction was carried out using the “Glitter (version 4.5)” software package. NIST SRM 610 synthetic glass (NIST, Gaithersburg, MD, USA) was used as an external standard, with 44Ca obtained from the XRF analyses as an internal standard. Precision and accuracy, both better than ±10% for concentrations at the ppm level, were monitored from repeated analyses of NIST SRM 612 and BCR-2g standards. The routine analyses consisted of a 1 min background acquisition and 1min of sample ablation. The signals of the following 45 masses were acquired during the analytical runs: 25Mg, 27Al, 29Si, 43Ca, 45Sc, 49Ti, 51V, 53Cr, 55Mn, 57Fe, 59Co, 60Ni, 63Cu, 66Zn, 71Ga, 85Rb, 88Sr, 89Y, 90Zr, 93Nb, 98Mo, 133Cs, 138Ba, 139La, 140Ce, 141Pr, 146Nd, 149Sm, 151Eu, 157Gd, 159Tb, 163Dy, 165Ho, 167Er, 169Tm, 173Yb, 175Lu, 177Hf, 181Ta, 185Re, 182W, 208Pb, 232Th, and 238U. 

### 2.7. Analysis of Biogenic Acids

The separation of organic acids by HPLC was carried out with an Adamas^®^ C18-X-Bond 5u (250 × 4.6 mm) column (Sepachrom, Rho, Italy) thermostated at 40 °C. The mobile phase used was a mixture of 10 mM copper (II) sulphate and 5 mM sulphuric acid (pH 2) in H_2_O at a flow rate of 0.5 mL/min. The injection volume was 20 µL and the reading wavelength was 240 nm. Calibration curves were made with the standards of the different acids at different concentrations. Quantification was based on peak areas. The retention times are oxalic acid: 3.8 min; malic acid: 5.2 min; and citric acid: 8.6 min. 

### 2.8. Regolith Powder Particle Size Analysis

The regolith was crushed by an agata planetary ball-mill and a powder was formed. The particle size of the regolith powder was analysed by a Mastersizer3000^®^ (Malvern Panalytical, Malvern, UK). The instrument was set to analyse silicates (Particle Refractive Index: 1457; Particle Absorption Index: 0.01; water was used as dispersant). Three analyses were conducted: on the pulverized regolith sample (untreated sample, regolith at time 0), regolith sample treated with organic acids (treated sample), negative control sample (TA-N + regolith). The negative control was included to evaluate the impact of mechanical agitation within the liquid medium on the granulometric size of the regolith. This was performed with the purpose of attributing any alterations in granulometric size to the effects of the acid, rather than to mechanical erosion resulting from particle movement within the liquid.

## 3. Results

### 3.1. Acid Production Rates

The evaluation of the organic acid production rate through pH reduction highlighted both the ability of *A. tubingensis* to generate organic acids and the speed of production. This allowed the determination of the acidification phase duration in the spent-medium bioleaching process.

**Figure 1:**
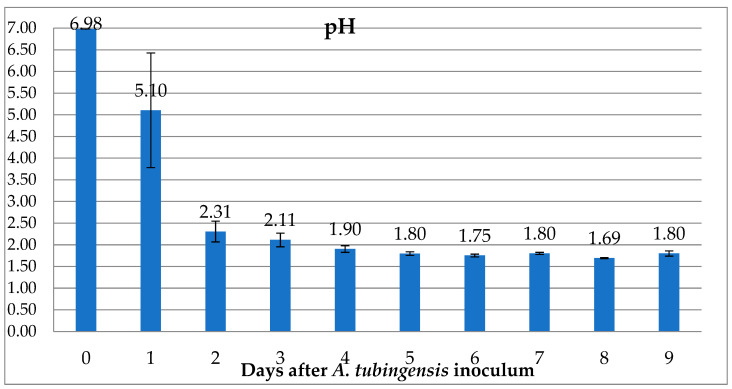
The graph displays the average pH decrease and the standard deviation in TA-N medium following the growth of the fungus.

The ability of *A. tubingensis* to lower the pH of the growth medium in a few days has been demonstrated. As evident from Figure 1, the results show a rapid decrease in pH values starting from an average value of 6.98 on day 0 to a value of 1.8 on day 9. These data indicate that the greatest reduction occurs rapidly between day 0 and day 2 (a decrease of −4.67 units). On day 3, two flasks reached a lower value; however, the average remained higher (2.11). On day 4, the average reached a pH value of 1.9, but one flask still maintained a value of 2. Finally, on day 5, the average value reached 1.8 with the less acidic flask having a value of 1.84. After the fifth day, there was no further significant decreases in pH, which stabilized around a value of 1.76.

### 3.2. Siderophores Production

The blue CAS test was conducted to assess whether the *A. tubingensis* strain could produce siderophores.

**Figure 2:**
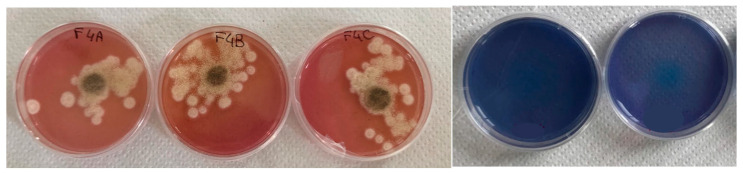
Left: The medium is completely red in all three plates surrounding the fungal inoculum of *A. tubingensis* producing siderophores (F4A-C represent 3 experimental replicates). Right: The control plates without inoculum have maintained the blue colour.

The test yielded positive results; despite the slight toxic effect of the CAS-assay, the *A. tubingensis* fungus was able to grow with three small colonies in each plate containing blue CAS_SAB. As shown in Figure 2, the colour of the plate rapidly changed around the three colonies from blue to red, demonstrating the presence of siderophores. The two control plates without inoculum showed no colour change.

### 3.3. Acid Production and Variation during Spent-Medium Bioleaching Process

The variation in acidity and composition of the acid medium used in the spent-medium bioleaching process are reported here. First, the pH change in the growth medium during the acidification step is described. Subsequently, the alteration of the liquid medium during the contact phase between the leaching solution and the regolith simulant is described. The changes in the mixture was detected both through pH evaluations and the quantification of organic acids by HPLC.

The organic acid analysis was conducted using the HPLC method to detect the presence of oxalic, malic, and citric acids. The presence of acids was investigated in experimental samples at three different stages: after the growth phase of *A. tubingensis*; after contact between the acids and regolith (acid after treatment); and finally, after leaving the acids not in contact with regolith for 3 weeks under the same experimental conditions (experimental control condition). The reported values in Table 1 represent the average of the analyses performed on two replicates for each experimental condition.

The acid composition in the control samples showed no statistically significant variations compared to the acid produced by the fungus after 5 days (Table 2). 

The acidity of the culture medium was also evaluated by measuring the pH. At the starting time, the TA-N medium had a pH value of 6.98; after 5 days of fungal growth, it dropped to 1.8 (−5.18). After 21 days of regolith treatment, it reached a value of 3.38 ± 0.02. The control sample did not substantially change in acidity, reaching a value of 1.82 ± 0.01.

The increase in the pH of the acidified medium, along with alterations in acid composition, becomes evident solely within the specimens wherein an interaction has occurred between the Mars regolith simulant and the acid solution. Consequently, the observed modifications may plausibly be ascribed to the interplay between the simulant and the acid leaching solution.

### 3.4. Fungal Activity Effect on the Regolith Powder Particle Size 

**Figure 3:**
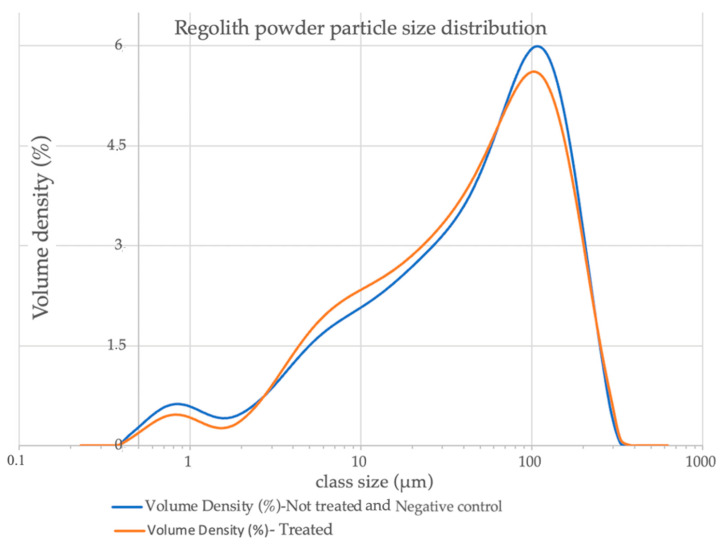
The graph displays the percentage volume of particles belonging to different size classes. The results are shown for the treated and untreated samples. The initial sample, after mechanical crushing, had a size range between 0.3 and 370 µm. The negative control sample perfect overlaps with the Not treated sample, so they are shown with the same blue line.

Particle analysis was conducted on the samples before and after treatment, as well as on the negative control. Figure 3 shows the values reported by the instrument and compares both the untreated sample and the negative control with the average of the treated samples. 

The blue line shows the particle size of the negative control sample (TA-N + regolith) and his trace perfect overlaps with the trace of the untreated sample (also blue line). The regolith of the negative control sample was immersed in liquid (TA-N) without the presence of acids and then underwent the same mechanical effect of rotating agitation at 120 rpm. Despite this, it did not show any changes to the particle size distribution, resulting in a similar profile as the untreated sample (as demonstrated by the overlay of the lines). The granulometric modification of the treated sample indicates that the biomining process simultaneously induces bio-weathering of the treated rock powder.

### 3.5. Fungal Action on Regolith Geochemical Composition

**Figure 4:**
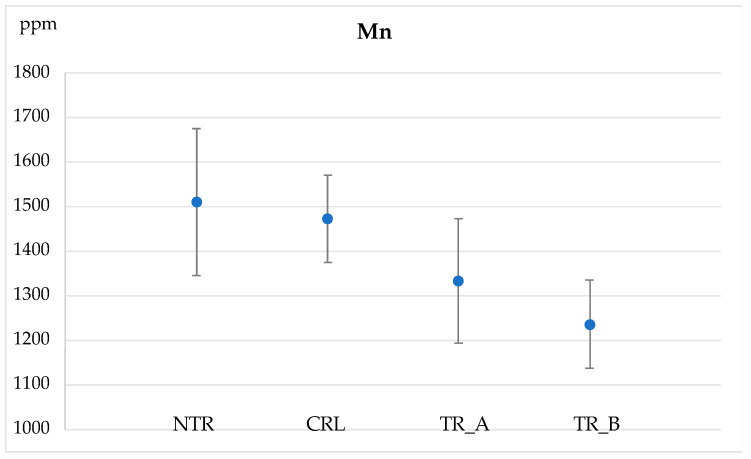
The graph displays the mean ppm values evaluated by the LA-ICP-MS instrument for the element Mn and relative standard deviations. Results are reported for NTR (untreated); CLR (control); TR_A and TR_B (the two experimental samples).

Four samples were analysed, with seven separate analyses performed on each sample. The concentrations are reported as average and standard deviations in Table 3. We analysed one untreated sample (NTR, regolith), one control sample (CRL, regolith in non-acidified TA-N), and two treated samples TR_A and TR_B (regolith in acidified TA-N). The two treated samples were subjected to the same experimental conditions. For most elements, analyses of NTR, CRL and TR_A and TR_B provided similar concentrations. The only exception was represented by Mn, which had lower concentrations in both treated materials compared to the original regolith (NTR) which was similar to the control sample (CRL) (Table 3; Figure 4).

## 4. Discussion

As evidenced by Figure 1, the evaluation of the pH reduction confirms the production of organic acids by *A. tubingensis* within the TA-N medium. This result is consistent with what has been reported in the literature for the *Aspergillus* genus and for the *A. tubingenesis* species [15,38]. At the same time, this experiment investigated the ability of this medium to stimulate the production of organic acids in the fungal strain under examination. 

This preliminary evaluation of the rate of organic acid production has led to identification of day 5 as the best day to conduct liquid media acidification. In fact, on day 5, all values were below a pH of 2, averaging around a value of 1.8. Days 3 and 4 had too much variability to be considered optimal for acid production, while days 6 to 9 show an average value of 1.76. 

The results obtained before the fifth day are not entirely reliable, from the perspective of industrial implementation of the technique under study. Additionally, waiting for a period longer than 5 days does not produce further reductions in pH values. This preliminary evaluation was considered important to optimise the acid production process, which is regarded as a crucial step in aligning the technology with the requirements of the industrial sector [39].

After determining the acidification rate, the strain’s ability to produce siderophores was also evaluated. As recorded in the literature for this species [10], the fungal strain of *A. tubingensis* was isolated and added to the collection of the mycology laboratory is able to produce siderophores. The test had a clearly positive result (Figure 2) with the colour of the medium turning from blue to red around all the colonies during fungal growth. 

The concentration of acids in the mixture, measured in mg/mL using the HPLC technique, decreased significantly after treatment (Table 1). The acids identified in this study are known to have the ability to form metallic organic salts with the ions present in the solution [40]. 

The formation of salts, as evidenced by the reduction in oxalic acid and citric acid content, serves as a positive indicator of the presence of metals in the bioleaching solution. Additionally, the formation of salts confirms the occurrence of complexolysis, which is the second essential reaction in the biomining process that follows acidolysis [17]. 

In conclusion, the observed changes in acid concentrations provides a reliable indication of the complexolysis process, serving as a crucial step in the overall extraction of metals from the rock substrate through fungal biomining.

During the biomining phase, the rock undergoes watering and the average particle size decreases (Figure 3). This is evident in the higher-size-class particles, which decreased, and the intermediate-size-class particles increased. The particles from the untreated sample and negative control sample (TA-N + regolith) have identical size distribution curves. The treated regolith shows a decrease in particles in the size class of 100 micrometres with an increase in the size class of 10 micrometres compared to the untreated sample. These results suggest that organic acids in the solution have induced a reduction in the grain size of the samples. This reduction in grain size could be associated with the leaching process for some trace metals [41]. 

The loss of Mn in the treated samples (Table 3; Figure 4) is clearly evident and suggests that Mn is more soluble compared to other metals in the acid solution. Although the concentration decrease was small and not always resolvable relative to the standard deviation of the untreated material, other elements (i.e., Pb) showed slightly depletions after the leaching process. Hence, we speculate that at the end of the process, Mn was the main metal to be bonded with the acids.

## 5. Conclusions

This study investigated the capabilities of a fungal strain belonging to the species *A. tubingensis* to extract metal from ores. The results showed that the strain could produce abundant amounts of organic acids in an exceptionally short time frame (<5 days). This acid production rapidly decreases the pH of the culture medium, making it suitable for bioleaching once it is separated from the fungus.

The fungal strain also tested positive in the blue CAS assay indicating that it possesses siderophores, a class of molecules that can mobilize metals within solid matrices. The release of siderophores can be beneficial in biomining processes as it induces a greater movement of metal ions, bringing them from the rock matrix into solution in the acidified water.

Finally, the experimental work focused on quantifying the biomining process on a Martian regolith simulant from different aspects. 

In all the tests, the untreated sample and the negative control behaved similarly, demonstrating that the absence of fungal organic acids and siderophores does not induce changes in the composition of the Martian regolith simulant. The regolith exposed to the fungal acids, on the other hand, underwent changes compared to the negative control and the untreated sample. The pH of the solution increased, and this process is attributable to the chemical phenomenon of acidolysis. Changes in the composition of the acid content of the liquid matrix (oxalic acid: −62%; citric acid: −38%), consistent with the increase in pH, were recorded by the HPLC analysis. The disappearance of these elements is attributable to the complexolysis phenomenon, able to cause the precipitation of metals into insoluble organic metal salts of oxalic and citric acids.

The bioleaching treatment, as determined by the analysis of particle size distribution, also induced bio-weathering. The samples treated for 21 days showed a decrease in the measurable particle size.

Finally, changes in geochemical compositions were only noted for Mn, whereas all the other elements did not undergo statistically significant decreases. The lack of decrease in the other elements contrasts the results obtained for Mn and may be likely attributed to the precipitation of organic salts containing metals formed during the complexation process. These salts might have been deposited within the analysed powder, underestimating the bioleaching process.

The results produced by this work indicate that the *A. tubingensis* strain is suitable for biomining and that the rock simulating the Martian substrate is a suitable mining substrate for this biological technique.

The possibility of exploiting techniques with lower environmental impacts and lower energy costs, even on extra-terrestrial substrates, opens new horizons for research. Although additional research is currently required to assess other biological and physical parameters that could influence the biomining process in extra-terrestrial contexts, the utilization of heterotrophic microorganisms such as fungi may yield further advantages. Fungal microorganisms could be fed organic waste from the astronaut diet, contributing to the recovery of waste products and recycling of nutrients [42].

Despite the positive results obtained, it is advisable that future work should focus on improving the separation of regolith from the acidic substrate. Critical issues were encountered in the separation process between the treated rock and the leaching solution.

## Figures and Tables

**Table 1:** Concentration of acids before and after regolith treatment.

	After the Growth Phase of *A. tubingensis*	Acid after Treatment	Reduction
Malic acid	0.044318	0.046523	No reduction
Citric acid	0.879691	0.500552	−0.37 (−38%)
Oxalic acid	3.812416	1.479217	−2.37 (−62%)

**Table 2:** Concentration of acids before treatment and in experimental control condition.

	After the Growth Phase of *A. tubingensis*	Acid in Experimental Control Condition	Reduction
Malic acid	0.044318	0.053955	No reduction (<0.00)
Citric acid	0.879691	0.882064	No reduction (<0.00)
Oxalic acid	3.812416	3.146086	−0.66633 (−17%)

**Table 3:** Average values in ppm of the element Mn in the samples NTR, CRL, TR_A, and TR_B.

LA_ICP_MS Detection of Manganese (Mn55 in ppm)			
NTR	CRL	TR_A	TR_B
1511.094 ± 164.9260	1473.46 ± 97.79	1332.324 ± 139.63	1235.671 ± 98.62

## Data Availability

Not applicable.
